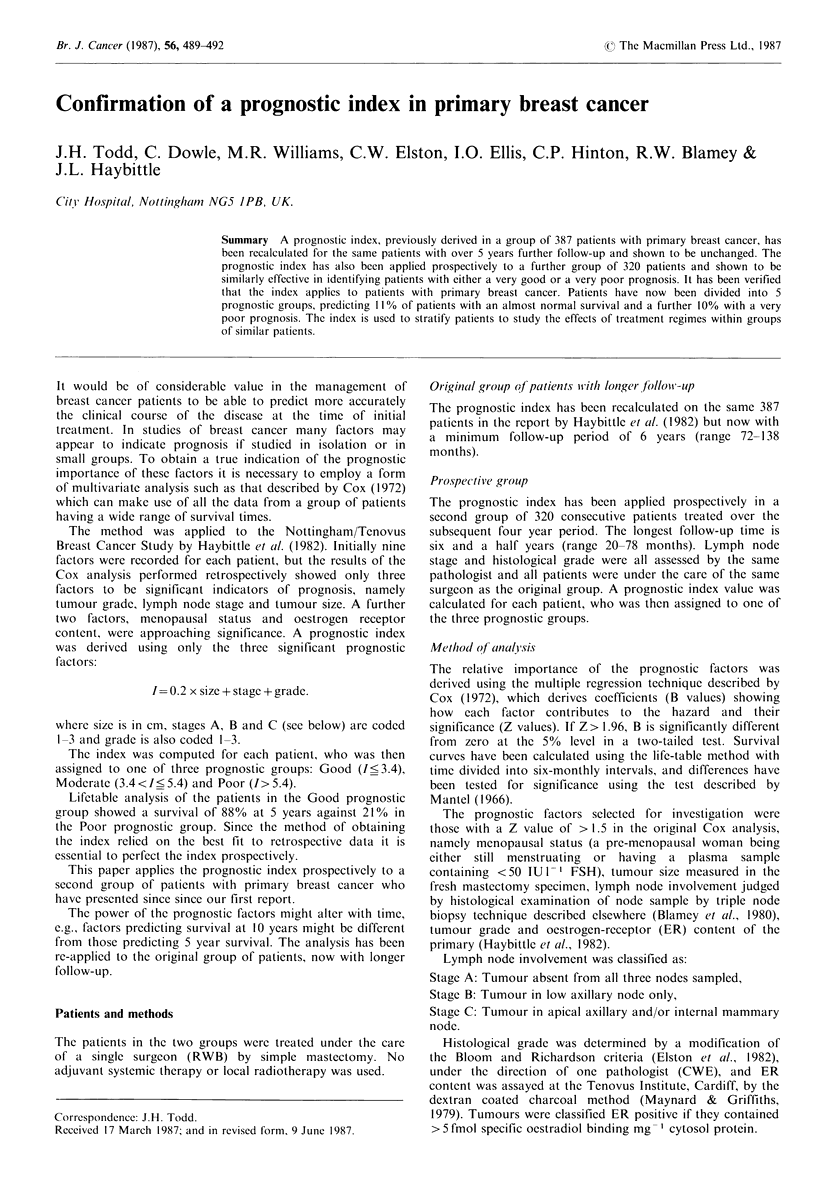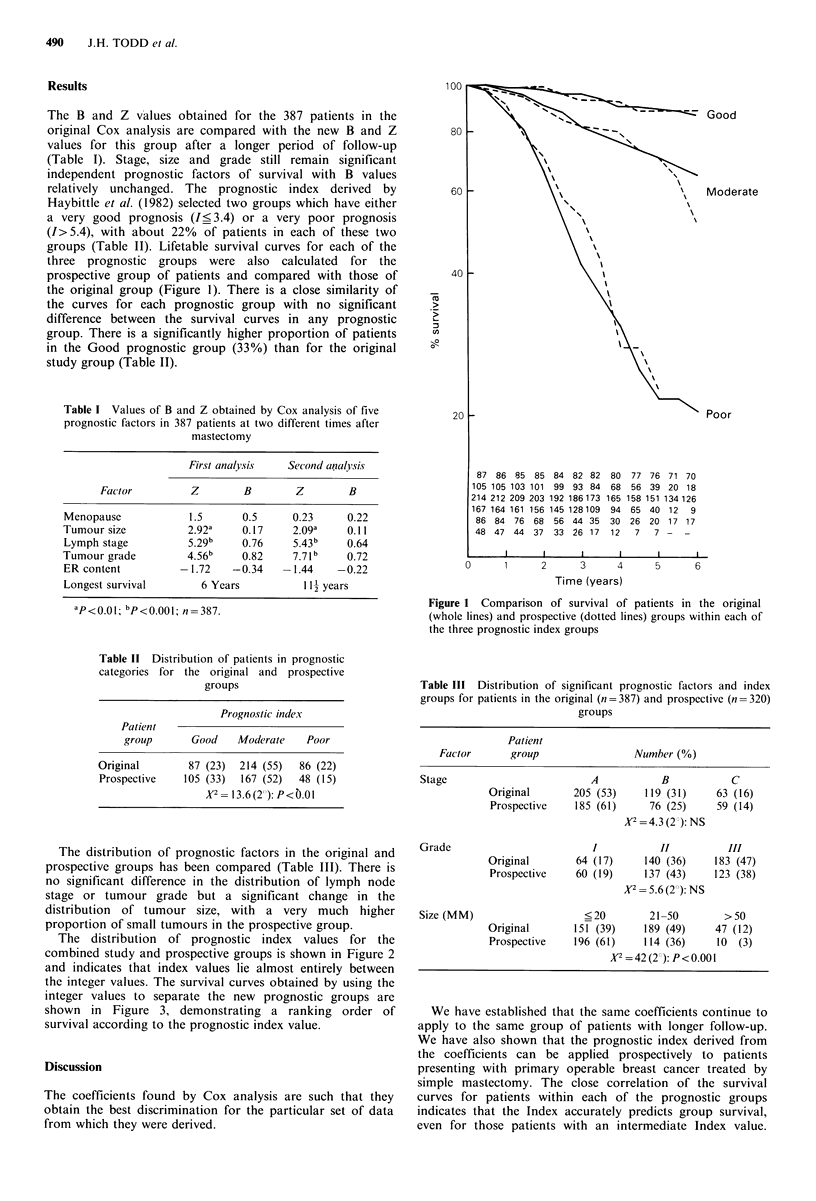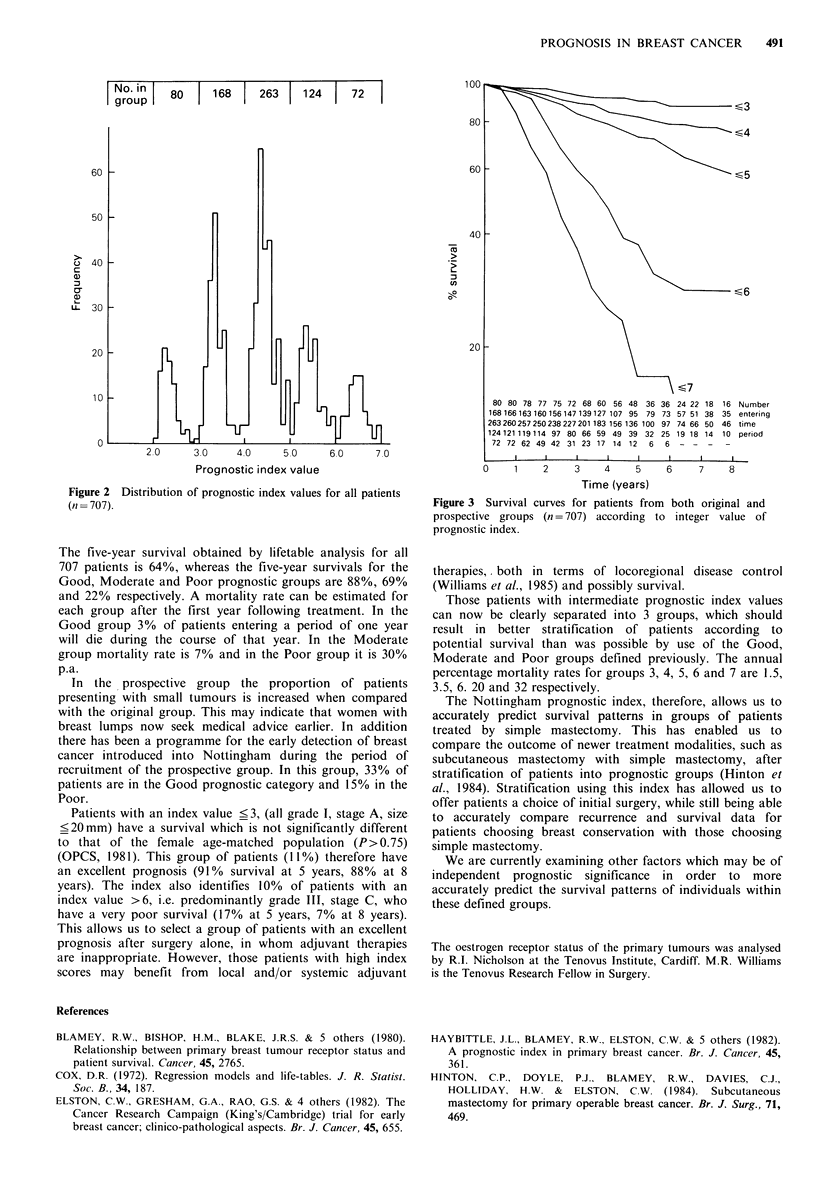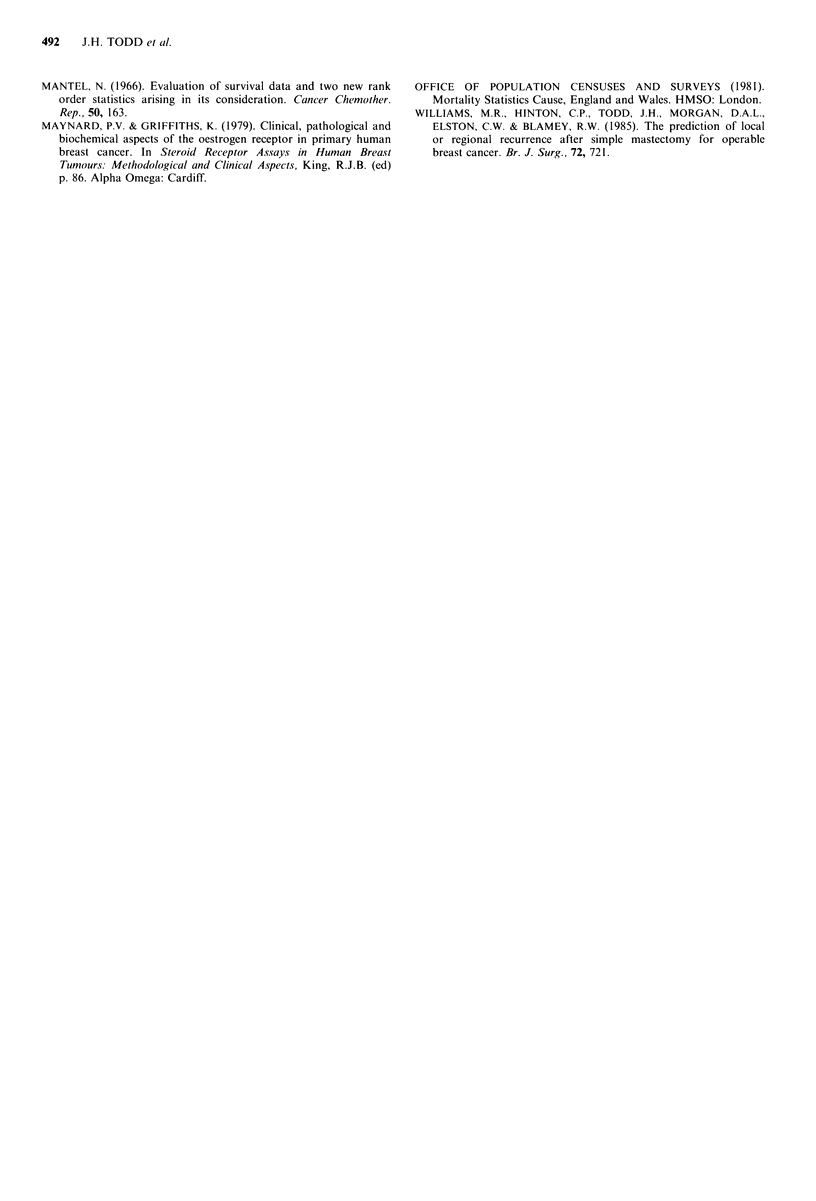# Confirmation of a prognostic index in primary breast cancer.

**DOI:** 10.1038/bjc.1987.230

**Published:** 1987-10

**Authors:** J. H. Todd, C. Dowle, M. R. Williams, C. W. Elston, I. O. Ellis, C. P. Hinton, R. W. Blamey, J. L. Haybittle

**Affiliations:** City Hospital, Nottingham, UK.

## Abstract

A prognostic index, previously derived in a group of 387 patients with primary breast cancer, has been recalculated for the same patients with over 5 years further follow-up and shown to be unchanged. The prognostic index has also been applied prospectively to a further group of 320 patients and shown to be similarly effective in identifying patients with either a very good or a very poor prognosis. It has been verified that the index applies to patients with primary breast cancer. Patients have now been divided into 5 prognostic groups, predicting 11% of patients with an almost normal survival and a further 10% with a very poor prognosis. The index is used to stratify patients to study the effects of treatment regimes within groups of similar patients.


*Br. J. Cancer* (1987), **56**, 489–492
© The Macmillan Press Ltd., 1987

### Confirmation of a prognostic index in primary breast cancer

J.H. Todd, C. Dowle, M.R. Williams, C.W. Elston, I.O. Ellis, C.P. Hinton, R.W. Blamey & J.L. Haybittle

*City Hospital, Nottingham NG5 1PB, UK.*

**Summary** A prognostic index, previously derived in a group of 387 patients with primary breast cancer, has been recalculated for the same patients with over 5 years further follow-up and shown to be unchanged. The prognostic index has also been applied prospectively to a further group of 320 patients and shown to be similarly effective in identifying patients with either a very good or a very poor prognosis. It has been verified that the index applies to patients with primary breast cancer. Patients have now been divided into 5 prognostic groups, predicting 11% of patients with an almost normal survival and a further 10% with a very poor prognosis. The index is used to stratify patients to study the effects of treatment regimes within groups of similar patients.

It would be of considerable value in the management of breast cancer patients to be able to predict more accurately the clinical course of the disease at the time of initial treatment. In studies of breast cancer many factors may appear to indicate prognosis if studied in isolation or in small groups. To obtain a true indication of the prognostic importance of these factors it is necessary to employ a form of multivariate analysis such as that described by Cox (1972) which can make use of all the data from a group of patients having a wide range of survival times.

The method was applied to the Nottingham/Tenovus Breast Cancer Study by Haybittle *et al.* (1982). Initially nine factors were recorded for each patient, but the results of the Cox analysis performed retrospectively showed only three factors to be significant indicators of prognosis, namely tumour grade, lymph node stage and tumour size. A further two factors, menopausal status and oestrogen receptor content, were approaching significance. A prognostic index was derived using only the three significant prognostic factors:

$$I = 0.2 \times \text{size} + \text{stage} + \text{grade}.$$

where size is in cm, stages A, B and C (see below) are coded 1–3 and grade is also coded 1–3.

The index was computed for each patient, who was then assigned to one of three prognostic groups: Good ($I \leqq 3.4$), Moderate ($3.4 < I \leqq 5.4$) and Poor ($I > 5.4$).

Lifetable analysis of the patients in the Good prognostic group showed a survival of 88% at 5 years against 21% in the Poor prognostic group. Since the method of obtaining the index relied on the best fit to retrospective data it is essential to perfect the index prospectively.

This paper applies the prognostic index prospectively to a second group of patients with primary breast cancer who have presented since since our first report.

The power of the prognostic factors might alter with time, e.g., factors predicting survival at 10 years might be different from those predicting 5 year survival. The analysis has been re-applied to the original group of patients, now with longer follow-up.

### Patients and methods

The patients in the two groups were treated under the care of a single surgeon (RWB) by simple mastectomy. No adjuvant systemic therapy or local radiotherapy was used.

Correspondence: J.H. Todd.

Received 17 March 1987; and in revised form, 9 June 1987.

*Original group of patients with longer follow-up*

The prognostic index has been recalculated on the same 387 patients in the report by Haybittle *et al.* (1982) but now with a minimum follow-up period of 6 years (range 72–138 months).

*Prospective group*

The prognostic index has been applied prospectively in a second group of 320 consecutive patients treated over the subsequent four year period. The longest follow-up time is six and a half years (range 20–78 months). Lymph node stage and histological grade were all assessed by the same pathologist and all patients were under the care of the same surgeon as the original group. A prognostic index value was calculated for each patient, who was then assigned to one of the three prognostic groups.

*Method of analysis*

The relative importance of the prognostic factors was derived using the multiple regression technique described by Cox (1972), which derives coefficients (B values) showing how each factor contributes to the hazard and their significance (Z values). If $Z > 1.96$, B is significantly different from zero at the 5% level in a two-tailed test. Survival curves have been calculated using the life-table method with time divided into six-monthly intervals, and differences have been tested for significance using the test described by Mantel (1966).

The prognostic factors selected for investigation were those with a Z value of $>1.5$ in the original Cox analysis, namely menopausal status (a pre-menopausal woman being either still menstruating or having a plasma sample containing $<50$ IU $l^{-1}$ FSH), tumour size measured in the fresh mastectomy specimen, lymph node involvement judged by histological examination of node sample by triple node biopsy technique described elsewhere (Blamey *et al.*, 1980), tumour grade and oestrogen-receptor (ER) content of the primary (Haybittle *et al.*, 1982).

Lymph node involvement was classified as:

Stage A: Tumour absent from all three nodes sampled,

Stage B: Tumour in low axillary node only,

Stage C: Tumour in apical axillary and/or internal mammary node.

Histological grade was determined by a modification of the Bloom and Richardson criteria (Elston *et al.*, 1982), under the direction of one pathologist (CWE), and ER content was assayed at the Tenovus Institute, Cardiff, by the dextran coated charcoal method (Maynard & Griffiths, 1979). Tumours were classified ER positive if they contained $>5$ fmol specific oestradiol binding mg$^{-1}$ cytosol protein.

### Results

The B and Z values obtained for the 387 patients in the original Cox analysis are compared with the new B and Z values for this group after a longer period of follow-up (Table I). Stage, size and grade still remain significant independent prognostic factors of survival with B values relatively unchanged. The prognostic index derived by Haybittle *et al.* (1982) selected two groups which have either a very good prognosis ($I \leqq 3.4$) or a very poor prognosis ($I > 5.4$), with about 22% of patients in each of these two groups (Table II). Lifetable survival curves for each of the three prognostic groups were also calculated for the prospective group of patients and compared with those of the original group (Figure 1). There is a close similarity of the curves for each prognostic group with no significant difference between the survival curves in any prognostic group. There is a significantly higher proportion of patients in the Good prognostic group (33%) than for the original study group (Table II).

**Table I** Values of B and Z obtained by Cox analysis of five prognostic factors in 387 patients at two different times after mastectomy

| | *First analysis* | | *Second analysis* | |
|---|---|---|---|---|
| *Factor* | *Z* | *B* | *Z* | *B* |
| Menopause | 1.5 | 0.5 | 0.23 | 0.22 |
| Tumour size | 2.92[a] | 0.17 | 2.09[a] | 0.11 |
| Lymph stage | 5.29[b] | 0.76 | 5.43[b] | 0.64 |
| Tumour grade | 4.56[b] | 0.82 | 7.71[b] | 0.72 |
| ER content | −1.72 | −0.34 | −1.44 | −0.22 |
| Longest survival | 6 Years | | 11½ years | |

[a]$P < 0.01$; [b]$P < 0.001$; $n = 387$.

**Table II** Distribution of patients in prognostic categories for the original and prospective groups

| *Patient group* | *Prognostic index* | | |
|---|---|---|---|
| | *Good* | *Moderate* | *Poor* |
| Original | 87 (23) | 214 (55) | 86 (22) |
| Prospective | 105 (33) | 167 (52) | 48 (15) |
| | $X^2 = 13.6(2°)$: $P < 0.01$ | | |

The distribution of prognostic factors in the original and prospective groups has been compared (Table III). There is no significant difference in the distribution of lymph node stage or tumour grade but a significant change in the distribution of tumour size, with a very much higher proportion of small tumours in the prospective group.

The distribution of prognostic index values for the combined study and prospective groups is shown in Figure 2 and indicates that index values lie almost entirely between the integer values. The survival curves obtained by using the integer values to separate the new prognostic groups are shown in Figure 3, demonstrating a ranking order of survival according to the prognostic index value.

### Discussion

The coefficients found by Cox analysis are such that they obtain the best discrimination for the particular set of data from which they were derived.

**Figure 1** Comparison of survival of patients in the original (whole lines) and prospective (dotted lines) groups within each of the three prognostic index groups

**Table III** Distribution of significant prognostic factors and index groups for patients in the original ($n = 387$) and prospective ($n = 320$) groups

| *Factor* | *Patient group* | *Number* (%) | | |
|---|---|---|---|---|
| Stage | | *A* | *B* | *C* |
| | Original | 205 (53) | 119 (31) | 63 (16) |
| | Prospective | 185 (61) | 76 (25) | 59 (14) |
| | | $X^2 = 4.3(2°)$: NS | | |
| Grade | | *I* | *II* | *III* |
| | Original | 64 (17) | 140 (36) | 183 (47) |
| | Prospective | 60 (19) | 137 (43) | 123 (38) |
| | | $X^2 = 5.6(2°)$: NS | | |
| Size (MM) | | ≦20 | 21–50 | >50 |
| | Original | 151 (39) | 189 (49) | 47 (12) |
| | Prospective | 196 (61) | 114 (36) | 10 (3) |
| | | $X^2 = 42(2°)$: $P < 0.001$ | | |

We have established that the same coefficients continue to apply to the same group of patients with longer follow-up. We have also shown that the prognostic index derived from the coefficients can be applied prospectively to patients presenting with primary operable breast cancer treated by simple mastectomy. The close correlation of the survival curves for patients within each of the prognostic groups indicates that the Index accurately predicts group survival, even for those patients with an intermediate Index value.

**Figure 2** Distribution of prognostic index values for all patients ($n = 707$).

**Figure 3** Survival curves for patients from both original and prospective groups ($n = 707$) according to integer value of prognostic index.

The five-year survival obtained by lifetable analysis for all 707 patients is 64%, whereas the five-year survivals for the Good, Moderate and Poor prognostic groups are 88%, 69% and 22% respectively. A mortality rate can be estimated for each group after the first year following treatment. In the Good group 3% of patients entering a period of one year will die during the course of that year. In the Moderate group mortality rate is 7% and in the Poor group it is 30% p.a.

In the prospective group the proportion of patients presenting with small tumours is increased when compared with the original group. This may indicate that women with breast lumps now seek medical advice earlier. In addition there has been a programme for the early detection of breast cancer introduced into Nottingham during the period of recruitment of the prospective group. In this group, 33% of patients are in the Good prognostic category and 15% in the Poor.

Patients with an index value $\leq 3$, (all grade I, stage A, size $\leq 20$ mm) have a survival which is not significantly different to that of the female age-matched population ($P > 0.75$) (OPCS, 1981). This group of patients (11%) therefore have an excellent prognosis (91% survival at 5 years, 88% at 8 years). The index also identifies 10% of patients with an index value $>6$, i.e. predominantly grade III, stage C, who have a very poor survival (17% at 5 years, 7% at 8 years). This allows us to select a group of patients with an excellent prognosis after surgery alone, in whom adjuvant therapies are inappropriate. However, those patients with high index scores may benefit from local and/or systemic adjuvant therapies, . both in terms of locoregional disease control (Williams *et al.*, 1985) and possibly survival.

Those patients with intermediate prognostic index values can now be clearly separated into 3 groups, which should result in better stratification of patients according to potential survival than was possible by use of the Good, Moderate and Poor groups defined previously. The annual percentage mortality rates for groups 3, 4, 5, 6 and 7 are 1.5, 3.5, 6. 20 and 32 respectively.

The Nottingham prognostic index, therefore, allows us to accurately predict survival patterns in groups of patients treated by simple mastectomy. This has enabled us to compare the outcome of newer treatment modalities, such as subcutaneous mastectomy with simple mastectomy, after stratification of patients into prognostic groups (Hinton *et al.*, 1984). Stratification using this index has allowed us to offer patients a choice of initial surgery, while still being able to accurately compare recurrence and survival data for patients choosing breast conservation with those choosing simple mastectomy.

We are currently examining other factors which may be of independent prognostic significance in order to more accurately predict the survival patterns of individuals within these defined groups.

The oestrogen receptor status of the primary tumours was analysed by R.I. Nicholson at the Tenovus Institute, Cardiff. M.R. Williams is the Tenovus Research Fellow in Surgery.

### References

BLAMEY, R.W., BISHOP, H.M., BLAKE, J.R.S. & 5 others (1980). Relationship between primary breast tumour receptor status and patient survival. *Cancer*, **45**, 2765.

COX, D.R. (1972). Regression models and life-tables. *J. R. Statist. Soc. B.*, **34**, 187.

ELSTON, C.W., GRESHAM, G.A., RAO, G.S. & 4 others (1982). The Cancer Research Campaign (King's/Cambridge) trial for early breast cancer; clinico-pathological aspects. *Br. J. Cancer*, **45**, 655.

HAYBITTLE, J.L., BLAMEY, R.W., ELSTON, C.W. & 5 others (1982). A prognostic index in primary breast cancer. *Br. J. Cancer*, **45**, 361.

HINTON, C.P., DOYLE, P.J., BLAMEY, R.W., DAVIES, C.J., HOLLIDAY, H.W. & ELSTON, C.W. (1984). Subcutaneous mastectomy for primary operable breast cancer. *Br. J. Surg.*, **71**, 469.

MANTEL, N. (1966). Evaluation of survival data and two new rank order statistics arising in its consideration. *Cancer Chemother. Rep.*, **50**, 163.

MAYNARD, P.V. & GRIFFITHS, K. (1979). Clinical, pathological and biochemical aspects of the oestrogen receptor in primary human breast cancer. In *Steroid Receptor Assays in Human Breast Tumours: Methodological and Clinical Aspects*, King, R.J.B. (ed) p. 86. Alpha Omega: Cardiff.

OFFICE OF POPULATION CENSUSES AND SURVEYS (1981). Mortality Statistics Cause, England and Wales. HMSO: London.

WILLIAMS, M.R., HINTON, C.P., TODD, J.H., MORGAN, D.A.L., ELSTON, C.W. & BLAMEY, R.W. (1985). The prediction of local or regional recurrence after simple mastectomy for operable breast cancer. *Br. J. Surg.*, **72**, 721.